# Predictive effects of foreign language listening emotions on listening achievement

**DOI:** 10.3389/fpsyg.2026.1809011

**Published:** 2026-04-23

**Authors:** Shuping Chen, Longhai Yu, Renjing Huang, Li Tian

**Affiliations:** 1 School of Humanities, Xinyu University, Xinyu, China; 2 Engineering Training Center, Xinyu University, Xinyu, China

**Keywords:** foreign language listening boredom scale, foreign language listening enjoyment scale, listening anxiety, listening emotions, positive psychology

## Abstract

**Introduction:**

While anxiety has been extensively studied, other emotions have remained largely unexamined in foreign language listening research, and their effects on listening achievement remain uncertain. This study aimed to investigate the predictive effects of anxiety, boredom, and enjoyment on listening achievement, while developing and validating foreign language listening boredom and enjoyment scales.

**Methods:**

Two sub-studies were conducted with three separate samples of Chinese English-as-a-foreign-language (EFL) university students (*N*₁ = 35; *N*₂ = 338; *N*₃ = 362). Based on open-ended data from Sample 1, Study 1 developed the preliminary Foreign Language Listening Boredom and Enjoyment Scales. These were then administered to Samples 2 and 3. Exploratory factor analysis with Sample 2 revealed a unidimensional structure for the boredom scale and a two-factor structure (private and social enjoyment) for the enjoyment scale. Confirmatory factor analysis with Sample 3 validated these structures, showing strong reliability (*α* = .89–.94) and multiple forms of validity. Study 2 collected listening achievement data from Sample 3, including IELTS and College English Test Band 4 listening scores and self-reported listening self-efficacy.

**Results:**

Regression analysis indicates that boredom consistently and negatively predicted listening achievement, whereas enjoyment appeared to have a positive effect; anxiety showed varying and context-dependent predictive influence.

**Discussion:**

The findings highlight the robust and distinct roles of multiple listening emotions in EFL listening success, supporting the need for emotion-sensitive listening pedagogy that fosters positive engagement and mitigates disengagement.

## 1 Introduction

Listening is an active, purposeful, and interactive process that depends on learners’ cognitive abilities and is influenced by affective factors (Vandergrift and Goh, 2012), including motivation, attitudes (Gardner et al., 1985), and emotions such as anxiety (Zhang, 2013). A large number of studies have mainly highlighted cognitive skills, such as aptitude and working memory in second language (L2) listening, whereas emotional factors have received comparatively less attention and have been examined primarily in relation to anxiety (e.g., Brunfaut and Révész, 2015; Vafaee and Suzuki, 2020; Zhang, 2013). However, individuals are not passive recipients of listening tasks or emotionless information processors driven solely by anxiety. Instead, listeners respond emotionally to listening activities and experience a range of emotions, encompassing both positive and negative emotional states (Dewaele and MacIntyre, 2014; Vandergrift and Goh, 2012). In addition, it is reasonable to assume that listeners bring their emotional resources to listening tasks and draw on positive emotions to energize themselves and adaptively engage with the linguistic challenges and cognitive demands of comprehension (Wang and MacIntyre, 2021). Grounded in control–value theory (Pekrun, 2006), which was introduced to L2 research during the rise of positive psychology (e.g., MacIntyre, 2016; MacIntyre et al., 2019), emotions are now viewed as integral to L2 listening because of their close connections with motivated listening behaviors (e.g., persistence and strategy deployment), cognitive functioning (e.g., attentional allocation), learner engagement, and self-regulatory processes.

In the present study, a comprehensive positive psychology perspective was adopted to examine both negative and positive emotions specific to L2 listening and their associations with listening outcomes. More specifically, we investigated the enjoyment, boredom, and anxiety experiences of Chinese university students who learn English as a foreign language (EFL). Listening achievement was operationalized using scores from the listening sections of the IELTS test and the College English Test Band 4 (CET4), as well as learners’ self-reported listening self-efficacy. Furthermore, foreign language listening boredom and enjoyment scales, two emotional variables that remain underexplored in L2 listening research, were created and psychometrically validated in the current study.

### 1.1 Emotions in L2 listening

Emotions are widely recognized to play a pivotal role in the listening process and exert a notable impact on listening behaviors and performance. Vandergrift and Goh (2012) posited in their influential metacognitive model of L2 listening that listening success is determined by the dynamic interaction between the listener, context, and text, with affective factors such as motivation and anxiety permeating and guiding the listener’s metacognitive processes of planning, monitoring, and evaluation. Wang and MacIntyre’s (2021) empirical study further confirmed that both listening anxiety and enjoyment are predictors of learners’ metacognitive awareness, with enjoyment enhancing such awareness and anxiety exerting an inhibitory effect on it. Shirvan and Taherian’s (2021) longitudinal study likewise confirmed that the positive emotion, foreign language enjoyment, has a substantial positive effect on maintaining learners’ listening motivational levels and engagement. It is plausible that learners with higher levels of listening enjoyment are more motivated to actively participate in and persevere with listening activities that demand high cognition.

L2 listening comprehension and evaluation are closely connected to learners’ emotional practices. In a seminal study, Goh (2000) studied learners’ think-aloud protocols and found that negative emotions such as frustration and anxiety frequently rise during online listening and are highly connected with cases of comprehension failure. This discovery aligns with the views of achievement emotion theory, which suggests that listening comprehension is a continuous process of self-monitoring, where learners’ perception of successful or unsuccessful comprehension prompts corresponding emotional responses—anxiety in the face of comprehension failure and a sense of satisfaction in the face of successful comprehension. Additionally, listening tasks are always located within social contexts (Rost, 2011), where interpersonal exchanges and classroom interactions can elicit social emotions such as embarrassment and empathy, particularly in feedback situations. In their foundational emotional model, MacIntyre and Gardner (1994) proposed that repetitive negative emotional practices, such as insistent listening anxiety, when accompanied by unproductive handling strategies (e.g., avoidance), can gradually develop into stable, trait-like emotional tendencies that serve as an enduring predictor of learners’ academic achievement and listening performances.

Grounded in Pekrun’s (2006) control–value theory of achievement emotions, this study adopts its comprehensive framework to understand learners’ emotional experiences. The theory posits that emotions are determined by two key appraisals: perceived control over a task and the subjective value attached to it. Different combinations of these appraisals give rise to distinct emotions. For instance, enjoyment emerges when learners perceive high control and high value; anxiety arises when tasks are important but perceived as uncontrollable; and boredom occurs when tasks are perceived as low in value or mismatched with learners’ abilities. Furthermore, the theory categorizes emotions along three dimensions: valence (positive vs. negative), activation (activating vs. deactivating), and object focus (activity vs. outcome). This framework has been widely applied in L2 research to explain the role of emotions in language learning processes and outcomes.

Applying this framework to the specific context of L2 listening, it becomes evident that listening tasks are particularly conducive to eliciting a range of achievement emotions due to their unique cognitive and social demands. From this perspective, L2 listening can be regarded as an emotionally demanding activity, due to the sequential and fleeting features of oral input and the cognitive effort required for processes like lexical segmentation and syntactic analysis (Field, 2010; Vandergrift and Goh, 2012), while also serving as a meaningful path for learners to advance linguistic ability, participate in authentic communication, and interact with interlocutors, teachers and peers. This highlights the multiple emotional dimensions existing in learners’ L2 listening engagement. Positive emotions are likely to arise when learners achieve effective comprehension and find the listening task appealing. By contrast, negative emotions are inclined to occur when learners struggle to decode the speech stream, fail to construct coherent meaning, or feel socially evaluated.

Drawing on the application of flow theory (Egbert, 2004) in positive psychology, positive emotions such as enjoyment are prone to occur when listeners view their abilities as matching the cognitive demands of the listening task. As outlined in control-value theory, positive emotions may arise when learners can effectively control a listening task that they believe is interesting or important (e.g., understanding a familiar topic). Conversely, learners are likely to experience negative emotions when they perceive important listening tasks as uncontrollable (e.g., accented or fast-paced speech). Boredom may also occur when tasks are perceived as unimportant or uninteresting, or when their difficulty level is seen as either excessively low or overly high (Li, 2021). Thus, the present study posits that L2 listeners will experience anxiety when exposed to high-speed input, unfamiliar accents or topics; listening tasks may elicit boredom if the content is repetitive, seen as irrelevant, or extremely above or under learners’ proficiency levels; and listening enjoyment is prone to occur when learners observe the activities as within their capability, inherently appealing, and worthwhile in terms of interpersonal connection or a sense of achievement.

While L2 listening emotions have received increasing theoretical attention, empirical research has largely focused on listening anxiety (Zhang, 2013). Initial evidence for the existence and potential effects of other emotional dimensions, nevertheless, can be found from qualitative inquiries into learners’ internal affective experiences. For example, from the analysis of learners’ interview data and diary entries, Graham (2006) discovered that L2 listening provokes a variety of emotional responses: learners described feelings of anxiety and frustration when they met comprehension difficulties, yet satisfaction and enjoyment arose when they successfully comprehended auditory input. This explicit empirical evidence of both negative and positive emotions related to listening performance offers a rationale for extending research focus outside anxiety to other emotions, such as enjoyment. Besides, boredom is commonly recognized in general language learning settings, where it is significantly connected with repetitive or low-cognitive-demand learning activities (Kruk and Zawodniak, 2020; Pawlak et al., 2020), signifying that similar listening tasks may elicit the same emotional responses.

Research into these emotions in the L2 listening field is at varying levels of maturity. Beyond confirming its role in the broader classroom context (Dewaele and MacIntyre, 2014), investigations into foreign language enjoyment have shifted to verifying its function as a significant positive predictor of listening-related constructs such as metacognitive awareness (Wang and MacIntyre, 2021). Conversely, while boredom in language learning has garnered growing research interest (Li, 2021; Li and Yang, 2025; Pawlak et al., 2020), its unique characteristics, antecedents, and consequences in the specific context of L2 listening remain mostly unexamined (Li et al., 2025b). Overall, these findings suggest that both enjoyment and boredom constitute prominent emotional experiences in L2 listening, yet they have scarcely been explored simultaneously or with equal investigative depth. This noteworthy gap in the existing literature thus demands a comprehensive examination into the extent to which these two emotions, alongside the more broadly studied listening anxiety, conjointly predict L2 listening achievement.

Listening emotions can be understood at two levels: trait and state. Grounded in educational psychology (Pekrun, 2006), trait emotions refer to learners’ enduring, consistent affective dispositions toward L2 listening formed over time in a specific learning context, such as general classroom instruction and routine listening practice. By contrast, state emotions are transient psychological responses prompted by specific task environments, such as a particularly difficult passage or an unfamiliar accent in a high-stakes test. Based on control–value theory, frequent experiences of a specific state emotion in similar listening contexts may progressively develop into a relatively permanent trait emotion, which in turn exerts more persistent influences on learners’ listening evaluations and performance (Pekrun, 2006). The current research focuses on these relatively stable trait emotions in the context of regular EFL classroom listening activities. We aim to investigate the trait tendencies of Chinese university students toward anxiety, enjoyment, and boredom that have developed through their prolonged engagement with listening tasks in instructional settings. It is important to acknowledge that different listening contexts, such as high-stakes testing, self-regulated learning, or classroom instruction, may elicit qualitatively different emotional profiles, as predicted by control–value theory’s emphasis on situational appraisals. For instance, test situations may heighten anxiety, while self-regulated learning might foster greater enjoyment or boredom depending on learners’ autonomy and task interest. Understanding these contextual variations is essential, and the present study is therefore situated within the specific context of regular EFL classroom listening instruction.

Different emotional categories (e.g., activating vs. deactivating, positive vs. negative) may exert distinct impacts on learners’ listening behaviors and performance. Therefore, it is highly important to explore these emotions and their unique contributions to listening outcomes. In accordance with the three-dimensional emotional classification proposed in the control-value theory (Pekrun, 2006), enjoyment is categorized as an activating positive emotion associated with task engagement, anxiety as an activating negative emotion linked to performance outcomes, and boredom as a deactivating negative emotion related to task involvement. An investigation into these three prevalent and theoretically distinct emotions enables a more holistic depiction of the affective landscape in L2 listening and its contributory role in listening achievement.

### 1.2 Second language listening anxiety, enjoyment, boredom, and achievement

It is widely acknowledged that anxiety has received the greatest scholarly attention among emotions examined in L2 listening research. Intending to move beyond general foreign language anxiety, Cheng (2017) argued that anxiety should be examined at the level of individual language skills and treated listening anxiety as a domain-specific emotional experience. Based on this perspective, Cheng (2017) designed and empirically validated a set of brief anxiety scales corresponding to the four language skills, including a dedicated measure for L2 listening anxiety. Psychometric analyses provided evidence for the reliability and construct validity of the listening anxiety scale, making it possible to investigate anxiety as a skill-specific variable in L2 listening. At present, however, such validated instruments are largely limited to anxiety, and comparable listening-specific measures for other emotions are still lacking. The absence of adequate methodological integration presents obstacles for investigations attempting to explain how distinct listening emotions affect listening achievements.

Research on the relationship between L2 listening anxiety and listening achievement has yielded inconsistent findings to date (Ji et al., 2022). Much research has verified a negative relationship between listening anxiety and listening achievement. For instance, Kim (2000) demonstrated that Korean EFL learners achieved poorer listening scores when they displayed higher degrees of listening anxiety. Recently, Liu and Lin (2025) observed that lowered levels of listening anxiety resulted in improved listening achievement. Conversely, other studies have not found significant or consistent relationships between listening anxiety and achievements. For instance, through structural equation modeling analysis, In’nami (2006) reported no significant effects of students’ listening test anxiety on L2 listening test scores, demonstrating that its impact may differ across contexts. Similarly, Liu (2016) found that the predictive effect of listening anxiety was non-significant among higher-proficiency learners, even though listening anxiety was negatively correlated with listening achievement. Likewise, Kim and Baek’s (2017) investigative analysis into the Korean elementary EFL learners through structural equation modeling demonstrated that listening anxiety did not uniquely predict L2 listening proficiency when cognitive and motivational factors were controlled. In sum, these conflicting findings show that listening anxiety may not consistently predict L2 listening achievement, highlighting the necessity of incorporating additional emotional variables to investigate listening achievement from a more comprehensive emotional perspective.

Compared with substantial studies on anxiety, a paucity of research has focused on positive emotions, such as enjoyment in L2 listening research. Recently, inspired by positive psychology, one study has arisen to investigate the contribution of listening enjoyment to listening achievement. Yang and Wu (2025) provided empirical evidence for a positive relationship between listening enjoyment and listening performance when they examined the mediating effects of enjoyment on the relationship between L2 listening self-efficacy, listening mindset, and listening comprehension. Nevertheless, this study assessed learners’ L2 listening enjoyment by adapting a six-item listening enjoyment questionnaire of Wang and MacIntyre (2021). This listening-specific measure remains to be validated regarding its factor structure and construct validity, which may raise concerns about the extent to which the adapted scale captures the domain-specific nature of L2 listening enjoyment.

In L2 listening research, boredom has been a mostly overlooked emotion compared with enjoyment and anxiety. Recently, two meta-analytic studies have consistently verified that boredom significantly and negatively impacts L2 achievement (Li et al., 2025b; Li and Yang, 2025). Notably, both studies explicitly recommended that the lack of boredom literature in listening-related L2 outcomes necessitates examining boredom in this specific L2 language skill. In spite of this, extremely scarce empirical research has directly investigated L2 listening boredom. More importantly, to date, no psychometrically validated scale has been developed to measure L2 listening-specific boredom, which further limits empirical research into its contribution to listening achievement. Overall, the inconsistent research results regarding listening anxiety, the arising but methodologically inadequate evidence on listening enjoyment, and the notable lack of empirical research and validated instruments on listening boredom call for further research into the role of L2 listening emotions in listening achievement.

## 2 The present study

Researchers and educators have been prioritizing identifying factors that enhance or hinder achievement in L2 learning. In spite of the increasing recognition of learner emotions in the field of L2 listening, limited empirical evidence has been provided pertaining to whether and to what extent multiple listening-specific emotions contribute to L2 listening achievement. Prior research on L2 listening anxiety has demonstrated inconsistent results regarding its influence on L2 learners’ listening outcomes. Additionally, emotions specific to listening, particularly enjoyment and boredom, have been comparatively underexplored.

To broaden understanding of this topic, this study analyzes how different listening-related emotions, specifically boredom, enjoyment, and anxiety, are linked to Chinese university EFL learners’ L2 listening achievement. Considering the shortage of well-validated tools for evaluating boredom and enjoyment in listening contexts, this study also aims to develop and validate two new scales to capture these emotional dimensions. Situated within the Chinese university EFL setting, this research aims to examine the following questions:

RQ1: To what degree do the Foreign Language Listening Boredom and Enjoyment Scales constructed in this study exhibit acceptable levels of reliability and validity?

RQ2: To what degree do foreign language listening anxiety, boredom, and enjoyment serve as predictors of L2 listening achievement?

Based on Research Question 2, the following hypotheses are proposed:

*H1:* Foreign language listening anxiety negatively predicts L2 listening achievement.

*H2:* Foreign language listening boredom negatively predicts L2 listening achievement.

*H3:* Foreign language listening enjoyment positively predicts L2 listening achievement.

### 2.1 Research design and participants

To examine these research questions, the present investigation consisted of two interrelated sub-studies. The first study aimed to develop and validate, in psychometric terms, newly created instruments measuring Chinese university EFL learners’ listening boredom and listening enjoyment. Study 2 focused on exploring the extent to which listening anxiety, boredom, and enjoyment function as predictors of L2 listening performance. An overview of the research aims, procedural steps, measurement tools, participant numbers, analytical approaches, and software employed across the two phases is provided in Table 1, with detailed descriptions reported in the sections that follow.

**Table 1:** Research design and procedure.

Phase	Procedure	Sample	Measures	Data analysis
Study 1: scale construction and psychometric evaluation	Item generation	Sample 1 (*N*_1_ = 35)	Open-ended prompts	Qualitative content analysis (Excel)
	Factor exploration	Sample 2 (*N*_2_ = 338)	Questionnaires (FLLES and FLLBS)	Exploratory factor analysis (SPSS 29.0)
	Factor validation	Sample 3 (*N*_3_ = 362)	FLLES and FLLBS questionnaires External criterion measures (AFLCAS, FLBS, CVFLES, FLLAS)	Confirmatory factor analysis (Mplus 8.3)
	Reliability and validity evaluation			Internal consistency: inter-item and item-total correlations, Cronbach’s *α* (SPSS 29.0) Construct validity: convergent, discriminant, and criterion-related validity (Excel; SPSS 29.0)
Study 2: Examining links between foreign language listening emotions and achievement	Collection of achievement data	Sample 3 (*N*_3_ = 362)	One IELTS listening test CET4 listening test Self-reported listening self-efficacy	Pearson correlation analysis (SPSS 29.0) Multiple regression analysis (SPSS 29.0)

*N* = sample size; FLLES = foreign language listening enjoyment scale; FLLBS = foreign language listening boredom scale; AFLCAS = abbreviated foreign language classroom anxiety scale; FLBS = foreign language boredom scale; CVFLES = Chinese Version of the foreign language enjoyment scale; FLLAS = foreign language listening anxiety scale; IELTS = international English language testing system; CET4 = college English test band 4. Convenience sampling was employed to recruit participants from a comprehensive university in central China. The participants were sophomore undergraduate learners attending required College English courses. Detailed demographic and background information for the three samples involved in the study is provided in Table 2. All participants were non-English majors drawn from a range of academic disciplines. During the data collection period, they had completed two semesters of compulsory College English courses and were enrolled in their third semester. English instruction was delivered through two 45-min class sessions per week (90 min in total), with listening and speaking activities systematically incorporated into the curriculum on a bi-weekly basis.

As outlined in Table 1, Study 2 focused exclusively on Sample 3 for examining the predictive relationships between listening emotions and achievement. This sequential approach was guided by the scale validation process: Sample 2 was used for exploratory factor analysis (EFA) to uncover the factor structures, while Sample 3 was used for confirmatory factor analysis (CFA) to validate these structures. Only after the scales’ factor structures were confirmed in Sample 3 did we proceed to examine their predictive effects on achievement, using the same sample to ensure that all analyses were conducted after the measurement model was established. Keeping the EFA sample independent from the main hypothesis testing follows recommended psychometric practices (Hair et al., 2010) and mirrors recent emotion scale development studies (e.g., Li et al., 2023a, 2023b).

The participants had received formal English education since primary school, with most beginning English learning in Grade 3 (approximately 8–9 years of age). During their first year at university, they all took the CET4, a nationwide standardized English proficiency examination for non-English majors in Chinese higher education. The CET4 evaluates a range of linguistic competencies, including writing, reading comprehension, listening comprehension, and translation. Their official CET4 scores, reported in Table 2 as background information, indicate that they had attained intermediate English proficiency, roughly equivalent to the B1+ level on the Common European Framework of Reference for Languages (CEFR). For Sample 3 only, an additional CET4 listening comprehension test was administered during the data collection period for Study 2. This separate test, which focused exclusively on listening comprehension, served as one of the outcome measures of listening achievement (see Section 4.2 for details).

**Table 2:** Participant demographics and background characteristics.

Sample	*N*	Female	Male	Mean age (SD)	Mean learning years (SD)	CET4 scores (0–710)
1	35	16	19	19.05 (0.80)	10.42 (1.46)	476.23 (96.77)
2	338	166	172	19.09 (0.67)	10.60 (1.36)	474.63 (89.92)
3	362	177	185	19.09 (0.82)	10.58 (1.39)	473.89 (87.49)

*N* = sample size; SD = standard deviation; CET4 = college English test band 4.

Ethical approval for the study was obtained from the university’s research ethics committee before data collection. Before participating in the research, each participant was informed of the procedures and purpose of the research and offered the written consent letter. Their participation in the research was voluntary, and their anonymity and privacy were guaranteed.

## 3 Study 1: Development and validation of foreign language listening enjoyment and boredom scales

### 3.1 Scale development

#### 3.1.1 Participants and data collection

The open-ended questionnaire was completed online by Sample 1 (*N*₁ = 35) in the university computer lab, providing all participants a consistent environment. This stage aimed to investigate learners’ emotional experiences in their L2 listening tasks, explore their observable behaviors and the corresponding origin of these emotional experiences, and extract themes from their responses. The Foreign Language Listening Boredom and Enjoyment scales were developed based on these extracted themes.

#### 3.1.2 Open-ended questions

Guided by Li et al.’s (2023a, 2023b) research approach, the following open-ended questions were designed to solicit participants to describe their listening emotions and identify their emotion sources: “*How do you feel when listening to English?”; “Why do you feel that way?” “Have you ever felt enjoyment/boredom during listening practice? When did it occur? Why?*”

#### 3.1.3 Data analysis and results

We conducted content analysis to investigate the perceived emotions of participants in the process of their listening practice and then extracted the indicators of foreign language listening boredom and enjoyment. More precisely, indicators of listening emotions were identified and extracted based on themes regarding emotions and their sources, and then the frequency of each emotion was calculated and reported.

*Frequencies of foreign language listening emotions*. A total of 78 descriptions of emotions were provided by the 35 participants, as participants were encouraged to report multiple emotions. As seen in Table 3, multiple emotional experiences were reported during participants’ listening practice, ranging from positive emotions (enjoyment, interest, immersion, happiness), neutral emotions (calmness), uncertainty related to their emotional situation, to negative emotions (anxiety, frustration, boredom). Foremost among these is enjoyment and then boredom, followed by anxiety and frustration.

**Table 3:** Descriptions of foreign language listening emotions and their frequencies.

Emotion	Description (exemplar keywords)	Frequency	Proportion
Enjoyment	Enjoyable, fun, happy, sense of achievement, immersed, interested.	24	30.8%
Boredom	Bored, tedious, distracted, monotonous, repetitive, uninteresting.	20	25.6%
Anxiety	Anxious, worried, afraid of not understanding, heart racing, mind blank.	15	19.2%
Frustration	Frustrated, upset, angry, feeling incompetent, wanting to give up.	9	11.5%
Excitement	Excited, eager, stimulated, challenging.	6	7.7%
Calmness	Calm, no particular feeling, not nervous, used to listening.	4	5.1%
Total		78	100%

Frequencies represent the number of coded emotional expressions identified from participants’ responses. Proportions are calculated based on the total frequency (*N* = 78). Exemplar keywords illustrate representative expressions associated with each emotion category.

#### 3.1.4 Generation of initial scale items

Drawing on relevant emotion theory, namely the control-value theory (Pekrun, 2006), as well as following prior L2 studies on foreign language learning boredom and enjoyment (Li et al., 2018; Li et al., 2023a, 2023b), the current study conceptualized the two constructs and created the preliminary item set.

First, aligning with theoretical constructs, item creation was guided by the three dimensions of achievement emotions in the control–value theory, i.e., valence, object focus, and activation. Boredom was conceptualized as a negative, activity-focused achievement emotion characterized by low activation, while enjoyment was described as a positive, activity-focused, activating achievement-related emotion. These theoretical obstructs were reflected in the design of the item sets of the Foreign Language Listening Boredom and Enjoyment Scales. For instance, items such as “*I feel tired and bored when the listening practice lasts too long*” and “*I feel irritated and bored when the listening material is too fast*” reflected the low activation associated with boredom while items such as “*I become fully immersed in listening practice and lose track of time*” and “*I feel happy when I can understand an English conversation*” captured the high activation associated with enjoyment.

Second, guided by these theoretical constructs, the control–value theory’s appraisal framework was reflected in developing items. In particular, the connections between learners’ control and value evaluation and their emotional experiences were represented in the construction of the items. For instance, the contribution of high perceived control to enjoyment was illustrated in the item “*I feel a sense of achievement when I understand the main idea after listening only once*,” whereas low perceived control related to boredom was exemplified in the item “*When the listening task is too difficult to understand, I just want to give up.*” Value evaluation was reflected in the item “*I am more willing to engage in listening when the material allows me to learn something new*.”

Last, the open-ended responses for listening enjoyment mirrored the appearance of the social/private aspect identified in prior research on general foreign language enjoyment (Li et al., 2018; Li et al., 2023a, 2023b), and thus, we incorporated it into the item creation of listening enjoyment. This approach was guided by evidence showing that listening enjoyment includes both personal dimensions (e.g., sense of accomplishment, immersion) and social dimensions (e.g., interactions with peers, supportive teacher feedback). Examples are the private enjoyment item, “*I enjoy listening practice when the material is interesting,*” and the social enjoyment item, “*I enjoy discussing listening answers with my classmates*.” By contrast, learners’ descriptions of listening boredom mainly focused on their individual responses to task characteristics and experiences of frustration, with little mention of social factors. Thus, the boredom scale primarily reflects individual experiences of disengagement and fatigue, as illustrated by items such as “*I find English listening practice boring*” and “*I become impatient if the accent in the listening material is unfamiliar.*”

Based on the themes extracted from the open-ended questionnaire responses, we referred to existing emotion scales (e.g., the Chinese Version of the Foreign Language Enjoyment Scale; the Foreign Language Learning Boredom Scale) and identified themes related to the antecedents and experiences of listening boredom and enjoyment as indicators for item generation. For listening boredom, we identified indicators such as “*finding the practice boring*,” “*feeling distracted by repetitive content*,” “*experiencing irritation toward fast speech*,” “*perceiving monotonous tone as dull*,” “*feeling tired from extended practice*,” “*being inclined to give up due to task difficulty*,” “*experiencing confusion from insufficient background information*,” “*losing patience with unfamiliar accents*,” “*finding exercises exam-oriented and meaningless*,” and “*growing weary of repetitive question types*.” For listening enjoyment, we extracted indicators such as “*feeling interested in listening practice*,” “*experiencing happiness from comprehension*,” “*enjoying practice due to interesting materials*,” “*gaining a sense of achievement*,” “*becoming immersed and losing track of time*,” “*anticipating teacher feedback*,” “*enjoying peer interaction*,” “*feeling pleased by teacher recognition*,” and “*increased engagement due to familiar topics, novel content, or teacher encouragement*.”

Building on these established indicators, developed a preliminary 10-item set of the Foreign Language Listening Boredom Scale (FLLBS) and an initial 12-item set of the Foreign Language Listening Enjoyment Scale (FLLES). Each item was rated on a five-point Likert scale, where “1” indicated strongly disagree and “5” indicated strongly agree.

Face validity evaluation. To ensure adequate face validity, a mutual review of the scales was conducted by the first and second authors, as well as two English professors of the participating university. The evaluation included whether each item was aligned with its related theoretical dimensions and if the phrasing was unambiguous, clear, and suitably tailored to university students. In addition, the English instructors were also asked to assess if the items aligned with the EFL listening curriculum at the university (e.g., textbook content, common task types). All disagreements were settled through discussion before we used the scales in the following phases of this research.

### 3.2 Scale validation I: Exploratory factor analysis of the proposed measures

We conducted an exploratory factor analysis (EFA) to identify the key constructs that constitute Foreign Language Listening Boredom (FLLB) and Foreign Language Listening Enjoyment (FLLE).

#### 3.2.1 Participants and instruments

Sample 2 (*N*₂ = 338) was administered online with the preliminary 10-item FLLB Scale and 12-item FLLE Scale. Data was collected in the computer laboratory of the university, where participants finished the questionnaires under supervision.

#### 3.2.2 Data analysis

We used SPSS 29.0 to carry out the EFA. First, the Kaiser–Meyer–Olkin (KMO) and Bartlett’s test of sphericity were used to guarantee the appropriateness of the data for factor extraction. The KMO value of the FLLB Scale was 0.966 (*p* < 0.001), and the FLLE Scale reached a KMO value of 0.947 (*p* < 0.001), which indicated that the datasets were appropriate for subsequent factor analysis (Field, 2013). Next, factors with eigenvalues exceeding 1 were extracted using principal component analysis, and a varimax rotation was applied to achieve a clearer factor structure.

#### 3.2.3 Factor structure

*Foreign language listening boredom scale (FLLBS)*. A single factor emerged from the EFA with an eigenvalue above 1, explaining 65.85% of the overall variance. As shown in Table 5, all item loadings ranged from 0.629 to 0.876, exceeding the recommended threshold of 0.40. Communalities ranged from 0.56 to 0.77. Item 9 (“*I feel that many listening exercises are only for exams and lack real meaning*”) was removed because its communality was slightly below 0.50 (0.40) and its semantic focus differed somewhat from the core experience of boredom (i.e., feelings of dullness and lack of motivation).

Accordingly, foreign language listening boredom was conceptualized as a unidimensional construct, referring to negative, low-activation, activity-related emotional and psychological states experienced during listening tasks. These include a general dislike of listening activities, fatigue caused by repetitive or monotonous content, frustration triggered by difficult tasks or unfamiliar accents, attention lapses arising from lack of interest, and the urge to avoid listening tasks.

*Foreign language listening enjoyment scale (FLLES)*. Two factors with eigenvalues greater than 1 emerged from the EFA, jointly explaining 66.58% of the variance (Factor 1: 56.15%; Factor 2: 10.43%). Table 4 shows that the item loadings varied between 0.616 and 0.829, all exceeding the suggested threshold of 0.40 (Hair et al., 2010). The communalities of the items ranged between 0.65 and 0.75. Item 9 (“All my classmates like doing English listening practice.”) was removed due to its low communality (<0.50) and the presence of cross-loadings. Its poor performance likely stems from its focus on perceived peer attitudes rather than learners’ own emotional experiences. Consistent with the open-ended responses in Sample 1, enjoyment was primarily described in relation to personal experiences and teacher interactions, with little mention of peer attitudes toward listening. The cross-loadings further suggested conceptual ambiguity, as the item appeared to tap into both enjoyment and boredom-related perceptions.

**Table 4:** Initial foreign language listening boredom scale.

Item No.	English	Chinese	Factor loadings
FLLBS1	I find English listening practice boring.	我觉得英语听力练习很无聊。	0.824
FLLBS2	I easily get distracted while listening if the content is repetitive.	听力时, 如果材料内容重复, 我很容易走神。	0.829
FLLBS3	I feel bored when the listening material is too fast.	当听力材料语速太快时, 我感到厌倦。	0.855
FLLBS4	I feel bored while listening if the speaker’s voice or tone is monotonous.	听力时, 如果说话者的声音或语调很单调, 我会感到厌倦。	0.852
FLLBS5	I feel bored when the listening practice lasts too long.	听力练习时间太长时, 我会感到厌倦。	0.810
FLLBS6^d^CFA	When the listening task is too difficult to understand, I just want to give up.	当听力任务太难, 完全听不懂时, 我只想放弃。	0.838
FLLBS7^d^CFA	I feel confused and bored while listening if there is insufficient background information.	听力时, 如果背景信息不足, 我会感到困惑和厌倦。	0.876
FLLBS8	I become impatient if the accent in the listening material is unfamiliar.	听力时, 如果口音不熟悉, 我会感到不耐烦。	0.829
FLLBS9^d^EFA	I feel that many listening exercises are only for exams and lack real meaning.	我觉得很多听力练习只是为了考试, 缺乏实际意义。	0.629
FLLBS10	I get tired of doing the same type of listening questions repeatedly.	重复做同一种类型的听力题让我感到厌烦。	0.745

FLLBS = foreign language listening boredom scale. ^d^EFA = items deleted during exploratory factor analysis; ^d^CFA = items deleted during confirmatory factor analysis. Factor loadings are based on the exploratory factor analysis.

The two factors identified were named Private Listening Enjoyment and Social Listening Enjoyment. Private Listening Enjoyment refers to activity-related positive emotions and psychological experiences during listening tasks, such as experiencing flow, deriving pleasure from comprehension or knowledge use, maintaining interest and confidence, and feeling satisfaction or achievement from personal growth. Social Listening Enjoyment reflects the positive emotions learners experience through interactions in the listening environment, including teachers’ supportive behaviors (e.g., encouragement, feedback, praise) and peers’ constructive engagement, which together create a motivating and collaborative learning atmosphere.

### 3.3 Scale validation II: Confirmatory factor analysis for scale validation

A confirmatory factor analysis (CFA) was performed to validate the factor structure obtained from the EFA and to further examine the scales’ construct validity.

#### 3.3.1 Participants and instruments

The revised 9-item Foreign Language Listening Boredom Scale (with FLLBS9 removed) and 11-item Foreign Language Listening Enjoyment Scale (with FLLES9 removed) were administered to Sample 3 (*N*₃ = 362) for validation.

#### 3.3.2 Data analysis

CFA was carried out using Mplus 8.3. Model fit was evaluated based on the following commonly accepted criteria, including: (1) *χ*^2^/df < 3; (2) a CFI value above 0.90; (3) a TLI value above 0.90; (4) an SRMR value below 0.08; (5) an RMSEA value below 0.08 (Hair et al., 2010).

#### 3.3.3 Factor structure and construct validity

**Figure 1:**
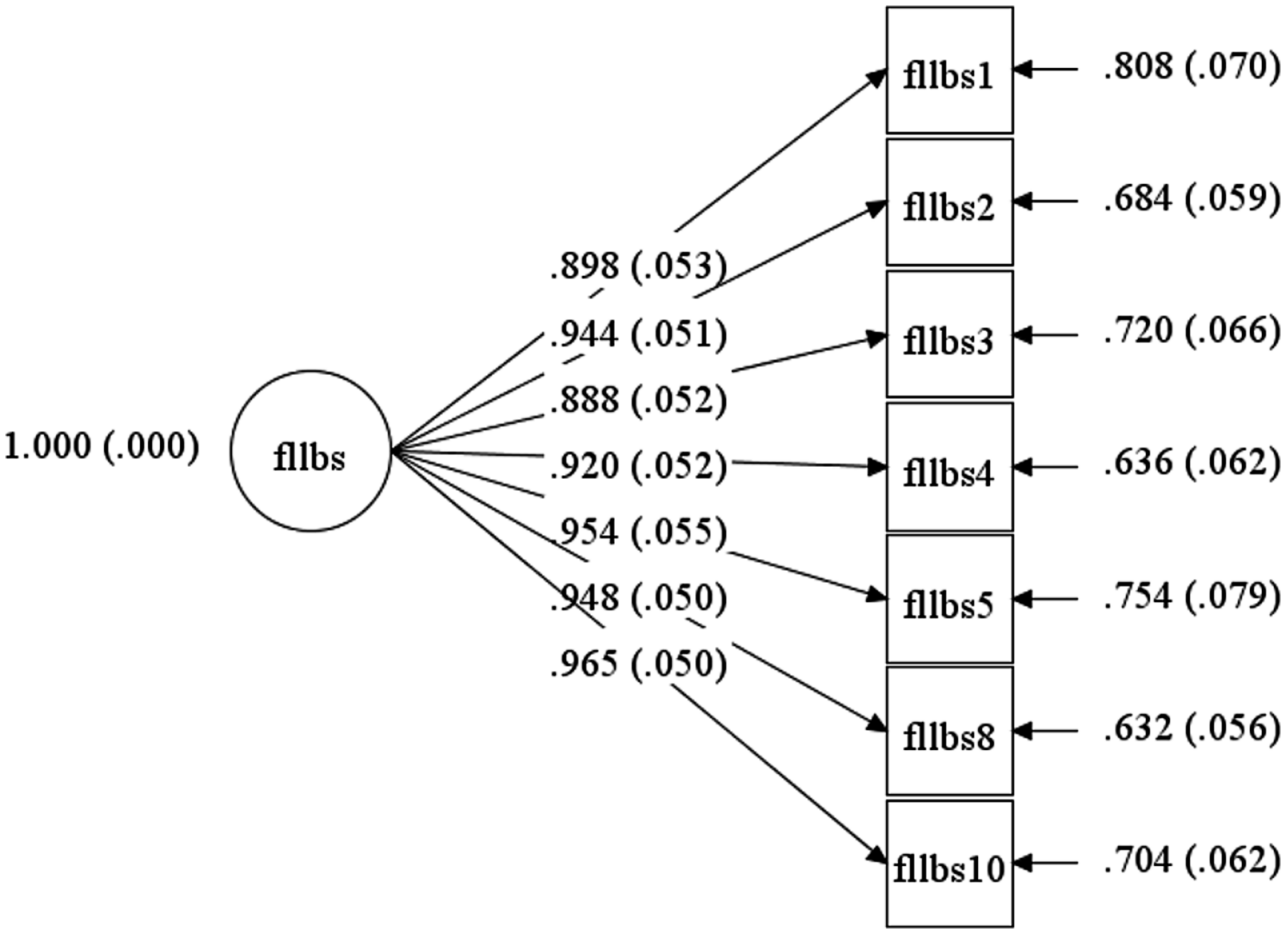
The single-factor model representing foreign language listening boredom.

**Figure 2:**
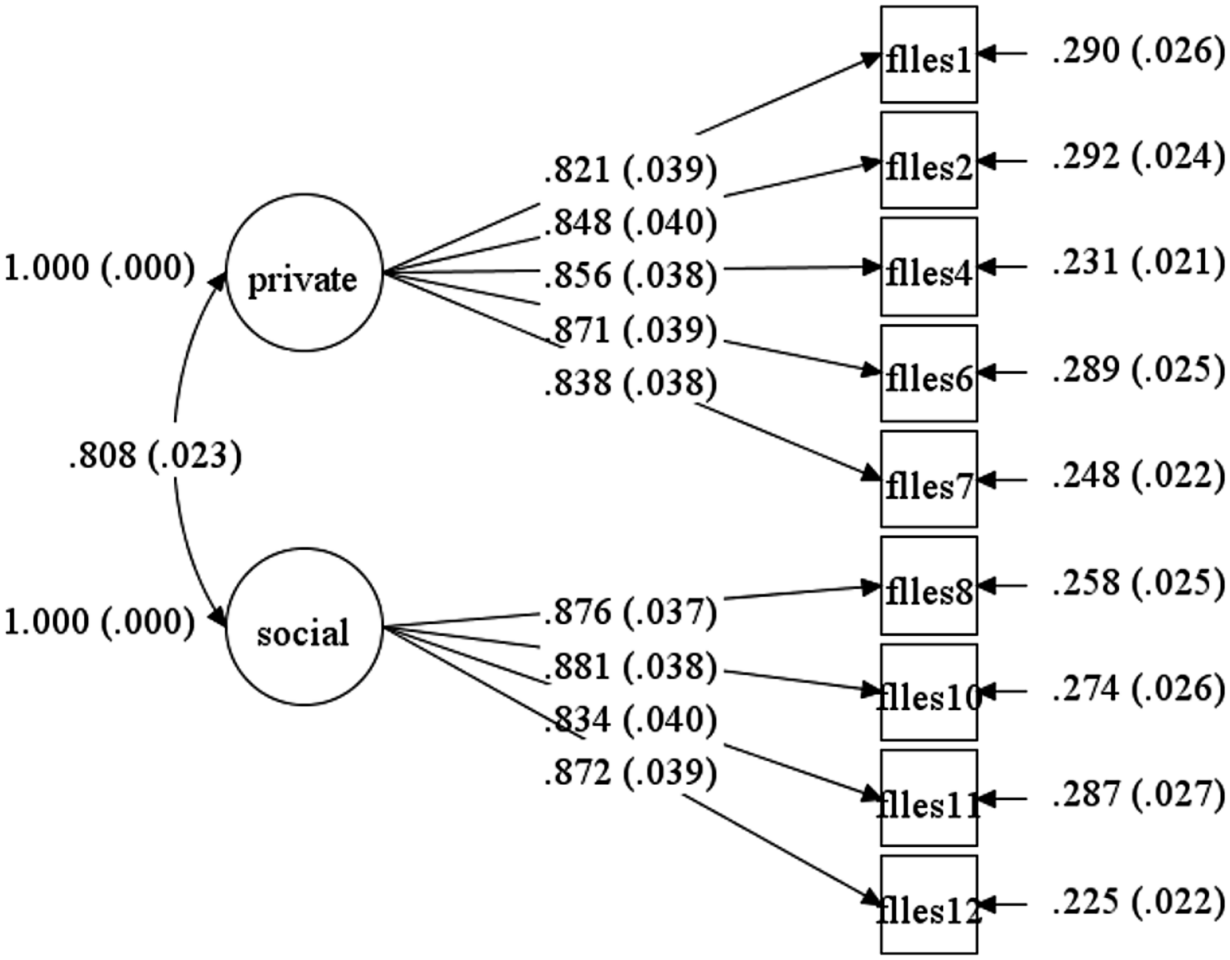
The dual-factor model representing foreign language listening enjoyment.

Model fit statistics are reported in Table 6, and standardized factor loadings are presented in Figures 1, 2. As shown in Table 6, the single-factor structure of the FLLBS and the hypothesized two-factor structure of the FLLES were confirmed in the main study following minor model refinements. Specifically, two items from the FLLBS (FLLBS6 and FLLBS7) were removed due to unacceptably low factor loadings (0.400 and 0.354, respectively) and poor reliability as indicated by their low R-squared values (0.160 and 0.126). For the FLLES, two items (FLLES3 and FLLES5) were removed due to significant cross-loadings on the alternative factor (M.I.s > 10), which compromised the discriminant validity of the two-factor structure.

All retained items exhibited standardized factor loadings exceeding 0.60 (see Figures 1, 2), supporting their adequacy for inclusion in the final scales. As illustrated in Figure 1, the two latent dimensions of the FLLES—private and social listening enjoyment—were highly and significantly correlated (0.808), indicating strong conceptual relatedness while maintaining discriminant distinction. Taken together, the CFA results support a unidimensional model for the FLLBS comprising seven items and a refined two-factor model for the FLLE consisting of nine items (see Tables 4, 5 for details of the final items).

**Table 5:** Initial foreign language listening enjoyment scale.

Item No.	English	Chinese	Factor loadings
Personal listening enjoyment subscale			
FLLES1	I feel happy when I can understand an English conversation.	当我能听懂一段英语对话时, 我感到很开心。	0.745
FLLES2	I am interested in English listening practice.	我对英语听力练习很感兴趣。	0.818
FLLES3^d^CFA	I become more engaged in listening when the topic is familiar to me.	如果听力话题是我熟悉的, 我会更投入。	0.765
FLLES4	I enjoy listening practice when the material is interesting.	听力练习时, 如果材料内容有趣, 我会很享受。	0.735
FLLES5^d^CFA	I am more willing to engage in listening when the material allows me to learn something new.	如果听力材料能让我学到新知识, 我会更愿意听。	0.808
FLLES6	I feel a sense of achievement when I understand the main idea after listening only once.	当我只听一遍就听懂主要内容时, 我感到很有成就感。	0.755
FLLES7	I sometimes become fully immersed in listening practice and lose track of time.	听力练习时, 我有时会完全沉浸其中, 忘记时间。	0.778
Social listening enjoyment subscale			
FLLES8	I always look forward to the English teacher’s feedback on my listening.	我总是很期待英语老师的听力反馈。	0.725
FLLES9^d^EFA	All my classmates like doing English listening practice.	我的同学都喜欢做英语听力练习。	0.616
FLLES10	I enjoy discussing listening answers with my classmates.	和同学一起讨论听力答案的过程让我感到愉快。	0.781
FLLES11	I feel pleased when the teacher acknowledges my progress in listening.	老师对我听力进步的肯定让我感到高兴。	0.829
FLLES12	The English teacher’s encouragement motivates me to participate in listening activities.	英语老师的鼓励让我更愿意参与听力活动。	0.712

FLLES = foreign language listening enjoyment scale. ^d^EFA = items deleted during exploratory factor analysis; ^d^CFA = items deleted during confirmatory factor analysis. Factor loadings are based on the exploratory factor analysis.

**Table 6:** Model fit statistics for the foreign language listening boredom scales and foreign language listening enjoyment (*N*_3_ = 362).

Scale	Factors	Items	*χ* ^2^	df	*p*	CFI	TLI	SRMR	RMSEA	90% CI
FLLB	1	9	27.83	27	0.420	0.999	0.999	0.023	0.009	0.000, 0.042
FLLB	1	7	15.34	14	0.356	0.999	0.998	0.016	0.016	0.000, 0.055
FLLE	2	11	79.54	43	0.001	0.978	0.972	0.035	0.048	0.031, 0.065
FLLE	2	9	42.94	26	0.020	0.994	0.991	0.018	0.042	0.017, 0.064

FLLBS = foreign language listening boredom scale; FLLES = foreign language listening enjoyment scale; *χ*^2^ = chi-square; df = degrees of freedom; CFI = comparative fit index; TLI = Tucker–Lewis’s index; SRMR = standardized root means square residual; RMSEA = root mean square error of approximation; CI = confidence interval. The 9-item and 11-item models represent the initial models, whereas the 7-item and 9-item models represent the final models after item deletion.

### 3.4 Scale validation III: Evaluation of scale reliability

The reliability of the FLLBS (7 items) and the FLLES (9 items), as confirmed in the preceding confirmatory factor analyses, was further evaluated through multiple indicators of internal consistency, including inter-item correlations, corrected item–total correlations, and Cronbach’s alpha. Reliability analyses were conducted using data from Sample 3 (*N* = 362).

Before subsequent parametric procedures, the distributional properties of the data were examined by inspecting item-level skewness and kurtosis values. For the FLLBS, skewness ranged from −0.15 to 0.08 and kurtosis from −1.07 to −0.87; for the FLLES, skewness values ranged from −0.07 to 0.08 and kurtosis values from −0.59 to −0.33. The data met the recommended criteria for normality, with skewness and kurtosis values within ±2 and ±7, respectively (West et al., 1995). Additionally, the distributions of the overall FLLBS score, the two FLLES subscales, and the overall FLLES score also met normality assumptions, with skewness ranging from −0.07 to 0.07 and kurtosis from −0.79 to −0.45. These findings showed that all the item-level, factor-level, and scale-level values displayed acceptable normality, supporting the use of parametric analyses in the following stages.

The inter-item correlations of FLLBS fell between 0.50 and 0.62, and FLLE inter-item correlations ranged from 0.55 to 0.77, exceeding the minimum 0.30 (Clark and Watson, 2016), indicating acceptable reliability results. The corrected item-total correlation values for the FLLBS were 0.67 to 0.72, and the corresponding values for FLLES were 0.77 and 0.80, which further confirmed adequate item discrimination. Strong internal consistency occurred as Cronbach’s alpha coefficients for the FLLBS, the overall FLLES, and its Private and Social Listening Enjoyment subscales all exceeded the acceptable threshold of 0.70, namely, 0.89, 0.94, 0.93, and 0.92, respectively.

### 3.5 Scale validation III: evaluation of scale validity

We assessed data from Sample 3 (*N* = 362) to conduct convergent validity, discriminant validity, and criterion-related validity of both the FLLBS and FLLES.

#### 3.5.1 Convergent validity

Convergent validity tests how the items targeting the same construct are correlated (Hair et al., 2010). Aligning with prior studies, we primarily used average variance extracted (AVE) and composite reliability (CR) to evaluate convergent validity (Fornell and Larcker, 1981; Hair et al., 2010). Thus, we utilized Excel to compute the AVE and CR values for both instruments based on the following formulas:


$$\mathrm{AVE}=\sum \lambda^{2}/n\mathrm{CR}={\left(\sum \lambda \right)}^{2}/[{\left(\sum \lambda \right)}^{2}+\sum \delta ]$$

where *λ* represents the standardized factor loading.

The AVE values for the FLLBS, the overall FLLES, and its two subdimensions (Private Listening Enjoyment and Social Listening Enjoyment) were 0.55, 0.73, 0.73, and 0.74, respectively. The CR values were 0.90 for the FLLBS, 0.96 for the overall FLLES, 0.97 for the Private Listening Enjoyment, and 0.92 for the Social Listening Enjoyment. All AVE values exceeded the suggested threshold of 0.50, and all CR estimates were above 0.70, which indicated that both scales achieved satisfactory convergent validity (Fornell and Larcker, 1981; Hair et al., 2010).

#### 3.5.2 Item- and factor-level evaluation of discriminant validity

We investigated discriminant validity across item- and factor-level measures. The item-level measure was conducted by applying an extreme-groups approach to assess the capability of individual items to discriminate among participants. We ranked the total scores of participants of Sample 3, and compared the lower and upper 27%. Independent-samples *t*-tests were carried out for each item across the two groups. The *t*-test results revealed that all items are significantly distinguished (*p* < 0.001), supporting adequate item discrimination.

To evaluate construct-level discriminant validity, the correlation between the two FLLES factors was analyzed. A correlation coefficient of 0.808 was observed between the two latent factors, falling below the widely accepted cutoff of 0.85 (see Figure 1; Hair et al., 2010).

#### 3.5.3 Criterion validity

Criterion validity concerns how well a target instrument shows systematic associations with other constructs that are theoretically related to it (DeVellis, 2016).

*Criterion measures*. In addition to completing the FLLES and FLLBS, participants in Sample 3 also responded to a listening-related anxiety scale as well as broader emotion measures. These measures were chosen as criterion variables due to their well-established theoretical connection to foreign language listening emotions in previous research (e.g., Li et al., 2018; Li et al., 2025a). All scales employed a 5-point Likert format, where 1 indicated “strongly disagree,” and 5 indicated “strongly agree.”

*Abbreviated foreign language classroom anxiety scale (AFLCAS)*. An abbreviated form of the Foreign Language Classroom Anxiety Scale, created by Horwitz et al. (1986) with 33 items, was used to assess general foreign language anxiety. This abbreviated version, originally containing eight items, was first used by Dewaele and MacIntyre (2014) and later underwent additional psychometric validation by Botes et al. (2022). After deleting two positively verbalized items that indicated inadequate factor loadings, Li and Li (2023) reduced and refined the scale to six items. This refined version was used in the current research as it has achieved reliability and factorial validity previously. Confirmatory factor analysis of this scale in the current sample revealed excellent fit: *χ*^2^(9) = 7.806, *p* = 0.554, CFI = 1.000, TLI = 1.002, RMSEA = 0.000 (90% CI [0.000, 0.053]), SRMR = 0.012., with standardized factor loadings ranging from 0.74 to 0.77. In this study, Cronbach’s alpha of the scale reached 0.89, demonstrating robust internal consistency.

*Foreign language boredom scale (FLBS)*. Li et al.’s (2023a, 2023b) foreign language Boredom Scale (FLBS), including 32 items, was used in this study to assess general foreign language boredom. This scale has been commonly used in subsequent studies, especially among university students (e.g., Li et al., 2025a). In the current sample, the 32-item seven-factor model also demonstrated excellent fit: *χ*^2^(443) = 457.456, *p* = 0.308, CFI = 0.993, TLI = 0.992, RMSEA = 0.009 (90% CI [0.000, 0.021]), SRMR = 0.036., with standardized factor loadings ranging from 0.41 to 0.5. In this research, strong internal consistency (*α* = 0.90) was shown in the scale.

*Chinese version of the foreign language enjoyment scale (CVFLES).* This study measured general foreign language enjoyment with Li et al.’s (2018) Chinese version of the Foreign Language Enjoyment Scale, a shortened version of the original Foreign Language Enjoyment Scale (Dewaele and MacIntyre, 2014). The Chinese version has been validated as a three-dimensional scale comprising Private Enjoyment (5 items), Teacher Enjoyment (3 items), and Atmosphere Enjoyment (3 items), and has demonstrated strong psychometric properties in Chinese EFL contexts (e.g., Li et al., 2018; Li et al., 2025a). A confirmatory factor analysis of the three-factor model in the current sample showed excellent fit: *χ*^2^(41) = 44.988, *p* = 0.309, CFI = 0.997, TLI = 0.997, RMSEA = 0.016 (90% CI [0.000, 0.041]), SRMR = 0.022, with standardized factor loadings ranging from 0.635 to 0.712. The three subscales demonstrated acceptable internal consistency (Cronbach’s *α* = 0.811, 0.676, and 0.729 for Private, Teacher, and Atmosphere, respectively). Given the established multidimensional structure and the lack of empirical support for a second-order global factor in our sample (second-order CFA: CFI = 0.879, RMSEA = 0.111, negative residual variance), we used the three subscale scores separately in the criterion-related validity analysis. This suggests that the CVFLES is best represented by its original three-dimensional structure (Li et al., 2018) rather than a single higher-order factor in our sample.

*Foreign language listening anxiety scale (FLLAS).* Listening-specific anxiety was assessed using the L2 Listening Anxiety Scale developed by Cheng (2017), which was designed to measure anxiety associated specifically with second language listening. The scale has demonstrated satisfactory validity and reliability in previous research with Chinese EFL university students and was therefore adopted in the present study. For this scale, confirmatory factor analysis in our sample showed acceptable fit: *χ*^2^(27) = 42.867, *p* = 0.027, CFI = 0.989, TLI = 0.986, RMSEA = 0.040 (90% CI [0.014, 0.062]), SRMR = 0.023, with standardized factor loadings ranging from 0.57 to 0.82. Internal reliability of the scale was high in the current sample (Cronbach’s *α* = 0.90).

*Data analysis and results*. To explore associations among FLLBS, FLLES, and their corresponding criterion measures, Pearson correlation analyses were conducted. As shown in Table 7, both scales were significantly associated with their criterion measures (ranging from −0.623 to 0.611). Specifically, Specifically, FLLBS was positively correlated with general foreign language anxiety (AFLCAS), general boredom (FLBS), and listening-specific anxiety (FLLAS), while FLLES showed negative correlations with these measures; both listening emotion scales also showed significant correlations with the three CVFLES subscales in the expected directions (all |*r*| > 0.50, *p* < 0.001).

**Table 7:** Relationships between foreign language listening boredom, enjoyment, and associated constructs (*N* = 362).

Scale	FLLES	AFLCAS	FLBS	FLLAS	CVFLES_Private	CVFLES_Teacher	CVFLES_Atmosphere
FLLBS	−0.466^***^	0.550^***^	0.611^***^	0.572^***^	−0.601^***^	−0.623^***^	−0.559^***^
FLLES	1	−0.548^***^	−0.585^***^	−0.544^***^	0.600^***^	0.520^***^	0.519^***^

FLLBS = foreign language listening boredom scale; FLLES = foreign language listening enjoyment scale; AFLCAS = abbreviated foreign language classroom anxiety scale; FLBS = foreign language boredom scale; CVFLES = Chinese version of the foreign language enjoyment scale; FLLAS = foreign language listening anxiety scale. Values represent Pearson correlation coefficients. ^***^p < 0.001.

## 4 Study 2: Predicting listening achievement from foreign language listening emotions

### 4.1 Participants and data collection

Listening achievement data were collected from Sample 3 (*N* = 362) approximately 2 weeks after the questionnaire administration. Listening achievement was treated as a multidimensional construct, including both objective performance and learners’ self-assessed listening proficiency, in line with prior studies highlighting the importance of combining self-assessment with standardized test results (Graham, 2003; Vandergrift and Goh, 2012). To capture this multidimensional construct, we collected data on three indicators of listening achievement, as detailed in in the following section.

### 4.2 Instruments for measuring foreign language listening achievement

To capture a comprehensive picture of listening achievement as a multidimensional construct, we employed three measures: the IELTS listening section, the CET4 listening section, and a listening self-efficacy questionnaire. The IELTS test represents an internationally recognized measure of academic listening proficiency, whereas the CET4 reflects nationally standardized, curriculum-aligned tasks familiar to participants. The combined use of these tests follows established practice in L2 listening research (e.g., Li, 2025; Yu and Zhao, 2014) and enables examination of whether the effects of listening emotions generalize across different test formats and proficiency benchmarks. Together with the self-efficacy measure, these instruments capture both objective performance and perceived competence, thereby strengthening the multidimensional assessment of listening achievement.

*The IELTS listening section (IELTS Listening).* We used the IELTS listening section as one of the indicators to assess participants’ English listening achievement. Comprising 40 items, the assessment includes multiple-choice, sentence completion, and matching formats, and measures understanding of various spoken English inputs (e.g., dialogues, monologues, and interviews). Each item received a score of 1, giving a total possible score between 0 and 40 in the current study. The scoring method demonstrated acceptable reliability in this sample, with Cronbach’s *α* = 0.87.

*The CET4 listening section (CET4 Listening).* The CET4 listening section served as the second measure of participants’ English listening achievement. As noted in Section 2.1, while all participants had taken the national CET4 exam during their first year of university, an additional CET4 listening comprehension test was administered specifically to Sample 3 during the data collection period for Study 2. This separate test, rather than their prior official CET4 scores, was used as an outcome measure. Comprising 25 multiple-choice questions, this section assesses understanding of main ideas, key details, and implied meanings in materials such as news reports, long conversations, and passages. Scoring was simplified without altering item weighting: items 1–15 received 1 point each, and items 16–25 received 2 points each, for a maximum total of 35 points. The scoring approach showed adequate internal reliability in this sample (Cronbach’s *α* = 0.74), meeting standard threshold criteria for reliability in a psychological educational assessment (Hair et al., 2010).

*Listening self-efficacy.* As a third indicator of listening achievement, participants’ self-efficacy in listening was assessed. Seven items adapted from the Questionnaire of English Self-Efficacy (Wang et al., 2014) were used to measure listening self-efficacy, focusing on Chinese EFL university students’ confidence in handling listening tasks like TV shows, English films, story comprehension, and radio programs. A 5-point Likert scale was used for participants to rate their confidence. Evidence from previous work with Chinese EFL university students supports the scale’s reliability and validity (Du and Man, 2022, 2023). Internal reliability of the listening self-efficacy scale was acceptable in the present study (Cronbach’s *α* = 0.89).

### 4.3 Data analysis

To investigate the predictive role of foreign language listening emotions on listening achievement, Pearson correlation analyses were first conducted to examine the associations between the three emotions (i.e., enjoyment, boredom, and anxiety) and each of the listening achievement indicators. Multiple linear regression was then carried out to examine the relationships. The models included the three listening emotions as independent variables, while participants’ IELTS listening scores, CET4 listening scores, and listening self-efficacy served as dependent variables. This strategy allowed for assessing the unique contribution of each listening emotion to objective listening test scores as well as self-assessed listening proficiency.

### 4.4 Results

Table 8 shows that negative associations were observed between listening achievement and foreign language listening boredom (FLLB, −0.513 to −0.604) as well as foreign language listening anxiety (FLLA, −0.345 to −0.456). Conversely, FLLE exhibited positive correlations with all listening achievement indicators (IELTS listening, CET4 listening, and listening self-efficacy), with effect sizes between 0.508 and 0.600.

**Table 8:** Correlations of foreign language listening emotions with measures of listening achievement.

Emotions	IELTS listening	CET4 listening	Self-efficacy
FLLB	−0.536^***^	−0.515^***^	−0.604^***^
FLLA	−0.345^***^	−0.330^***^	−0.456^***^
FLLE	0.508^***^	0.538^***^	0.600^***^

FLLB = foreign language listening boredom; FLLA = foreign language listening anxiety; FLLE = foreign language listening enjoyment; IELTS = international English language testing system; CET4 = college English test band 4. Values represent Pearson correlation coefficients. ***p < 0.001.

Guided by the observed correlation patterns, multiple regression analyses were performed to investigate how the three listening emotions predict the three indicators of listening achievement. Table 9 provides an overview of the findings. The regression models including FLLB, FLLA, and FLLE as simultaneous predictors were significant across all outcome measures (adjusted *R*^2^ = 0.370–0.491, *p* < 0.001). Specifically, FLLE positively predicted all three outcomes (*β*s = 0.352–0.423); FLLB negatively predicted all three outcomes (*β*s = −0.388 to −0.408). Interestingly, FLLA showed differential predictive patterns: it positively predicted CET4 listening achievement (*β* = 0.121, *p* = 0.027) but did not significantly predict IELTS listening achievement (*β* = 0.063, *p* = 0.257) or self-efficacy (*β* = −0.036, *p* = 0.462). For IELTS listening, FLLB (*β* = −0.408) and FLLE (*β* = 0.352) both contributed significantly. For CET4 listening and self-efficacy, FLLE (*β* = 0.423 and 0.394) and FLLB (*β* = −0.388 and −0.399) also showed significant effects.

**Table 9:** Combined contributions of foreign language listening emotions to listening performance (*N*₃ = 362).

Listening Achievement	*R* ^2^	Δ*R*^2^	*F*	Emotions	*B*	*β*	*t*	*p*	95% C.I.		Collinearity	
									Lower	Upper	Tol.	VIF
IELTS Listening	0.38	0.37	71.65^***^	FLLB	−0.48	−0.41	−7.80	***	−0.60	−0.36	0.64	1.57
				FLLA	0.05	0.06	1.14	0.25	−0.04	0.13	0.58	1.74
				FLLE	0.38	0.35	6.89	***	0.27	0.49	0.67	1.50
CET4 Listening	0.39	0.38	75.39^***^	FLLB	−0.38	−0.39	−7.49	***	−0.48	−0.28	0.64	1.57
				FLLA	0.079	0.12	2.22	0.03	0.01	0.15	0.58	1.74
				FLLE	0.38	0.42	8.36	***	0.29	0.47	0.67	1.50
Self-efficacy	0.49	0.49	117.08^***^	FLLB	−0.24	−0.40	−8.50	***	−0.30	−0.19	0.64	1.57
				FLLA	−0.02	−0.04	−0.74	0.46	−0.06	0.02	0.58	1.74
				FLLE	0.22	0.39	8.59	***	0.17	0.27	0.67	1.50

FLLB = foreign language listening boredom; FLLA = foreign language listening anxiety; FLLE = foreign language listening enjoyment; *B* = unstandardized coefficient; *β* = standardized coefficient; CI = confidence interval; Tol. = tolerance; VIF = variance inflation factor; Δ*R*^2^ = change in R-squared. Values represent results of multiple regression analyses. ***p < 0.001.

All 95% confidence intervals for significant regression coefficients excluded zero, indicating reliable effects. Multicollinearity was examined and ruled out (tolerance > 0.575, VIF < 1.741).

In summary, the regression results supported H2 and H3, indicating that listening boredom negatively predicted listening achievement and listening enjoyment positively predicted listening achievement across all three outcome measures. In contrast, H1 was not supported. Listening anxiety was not a consistent predictor, showing a positive effect on CET4 listening scores (*β* = 0.121, *p* = 0.027) but no significant effect on IELTS listening scores or listening self-efficacy.

## 5 Discussion

### 5.1 Establishing the soundness of FLLBS and FLLES

RQ1 aimed to assess the psychometric properties of the two listening-related emotion scales developed for this study: FLLBS and FLLES. The converging evidence obtained across a range of reliability and validity checks lends support to the adequacy of these instruments for capturing learners’ emotional experiences during foreign language listening.

Regarding scale structure and construct validity, results from both EFA (Section 5.2) and CFA (Section 5.3) supported a unidimensional structure for the FLLBS and a two-factor structure for the FLLES, representing private and social aspects of listening enjoyment. The two-factor structure demonstrates that both intrapersonal and interpersonal emotional experiences are involved in listening enjoyment. This result aligns with the previous perspectives of viewing L2 listening as an active meaning-constructing process during which learners’ cognitive processing arises and is reinforced by classroom interactions with teachers, peers, and listening activities (Field, 2010; Rost, 2011; Vandergrift and Goh, 2012). By comparison, the one-factor structure and the impact limitation of social interactions evidenced that listening boredom seems to be a brief intrapersonal emotion.

Evaluating the scales through a multi-indicator approach verifies the robust strength of the reliability and validity of the current FLLB and FLLE scales. Various metrics, including internal consistency estimates, the relationships between items, and item-total correlations (see Section 5.4), evidenced the scales’ reliability. Multiple angles were used to evaluate validity, namely convergent validity, discriminant validity at the item and factor levels, and criterion-related validity, as reflected in the links between the target listening emotions and theoretically correlated emotional constructs (see Section 5.5).

Notably, further investigation is necessary for these two scales to be generalized in contexts beyond this research. The two scales were designed and assessed among Chinese non-English-major university students whose listening practice happens predominantly in structured, curriculum-driven English learning situations. Previous research has argued that listening practice varies noticeably across teaching contexts and learner groups (Field, 2010; Vandergrift and Goh, 2012). To be specific, English learners who use listening for professional or academic objectives, such as university students employing L2 English in content-centric contexts, are likely to confront listening challenges differentiated evidently from those in standard foreign language classrooms. These differentiations necessitate a broader comparison between L2 listening, which is mainly utilized to acquire content knowledge, and foreign language listening, which is briefly used for language learning purposes. The difference across task requirements and learning purposes is robustly linked to learners’ emotional experiences (Pekrun, 2006). Therefore, further research is necessary to assess whether the two listening emotion sales apply across different learner groups in both second and foreign language contexts.

### 5.2 The differential predictive patterns of listening emotions

RQ2 examined whether the three various listening emotions (FLLB, FLLA, and FLLE) serve as predictors of Chinese university EFL learners’ listening achievement. The findings exhibit differential and subtle predictive extents, highlighting the emotional dimensions of L2 listening. Specifically, the results supported H2 and H3, with listening boredom negatively and listening enjoyment positively predicting listening achievement across all three outcome measures. In contrast, H1 was not supported: listening anxiety did not show the expected negative effect and appeared to have context-dependent influences, being positively associated with CET4 listening scores but not with IELTS listening scores or listening self-efficacy.

#### 5.2.1 The distinct role of listening anxiety

An especially thought-provoking finding relates to the distinct predictive effects of FLLA. Though the bivariate analyses indicated negative correlations between FLLA and all three listening outcome measures, the multiple regression models exhibited considerably different unique predictive effects of FLLA. Specifically, listening anxiety exerted a positive prediction on CET4 listening achievement (*β* = 0.121, *p* = 0.027) but did not serve as a significant predictor of either IELTS listening scores (*β* = 0.063, *p* = 0.257) or self-efficacy (*β* = −0.036, *p* = 0.462). These findings are reflections of the inconsistent effects of L2 listening anxiety reported in prior research on its contributions to listening achievement (Ji et al., 2022), showing the differing impact of anxiety across achievement measures and contexts.

The findings regarding the positive predictive effect of listening anxiety on CET4 listening scores are in contrast with the more common negative correlation reported in existing studies (e.g., Liu and Lin, 2025). However, this result aligns with the Yerkes–Dodson law (Yerkes and Dodson, 1908), a well-recognized model delineating an inverted U-shaped relationship between arousal and performance, which still influences optimal cognitive functioning research (Diamond et al., 2007). Scovel (1978) described this nonlinear effect in language learning as the distinction between facilitating and debilitating anxiety, which was highlighted in the recent syntheses of language emotion studies (e.g., Elahi Shirvan et al., 2024). Grounded by Pekrun’s (2006) control-value theory, it can be inferred that when learners perceive control over a challenging but familiar test, such as the CET4, their anxiety can promote concentration and prompt effortful engagement. Further support may be found in Cassady and Johnson’s (2002) meta-analytic study, which concluded that moderate test anxiety can facilitate performance under specific contexts. Meanwhile, the context-specific characteristic of emotional impacts offers further explanation. For the IELTS listening, its increasing perceived importance and its international and relatively unfamiliar diverse format may tempt anxiety beyond a helpful level, thus damaging cognitive behaviors. This interpretation can be evidenced in In’nami’s (2006) study, which found that adverse effects of anxiety may occur in less familiar test formats.

An alternative statistical explanation for the positive effect of listening anxiety on CET4 listening scores is a suppression effect (Martinez Gutierrez and Cribbie, 2021). In the multiple regression model, listening anxiety, boredom, and enjoyment were entered simultaneously as predictors. Because boredom and enjoyment share substantial variance with anxiety (see Table 7, *r* = 0.572 and −0.544, respectively), it is possible that anxiety acts as a suppressor variable. Specifically, anxiety may carry unique variance that is irrelevant to the outcome (e.g., disengagement or worry) and, when that irrelevant variance is partialled out by the inclusion of boredom and enjoyment, the remaining unique component of anxiety could show a positive relationship with listening performance. Although formal tests of suppression (e.g., comparing zero-order and partial correlations) were beyond the scope of the present study, the observed pattern where listening anxiety showed a weak negative zero-order correlation with CET4 listening (*r* = −0.330, see Table 8) but a positive regression coefficient (*β* = 0.121) aligns with a classic suppression pattern. This suppression effect would not necessarily imply that anxiety is “facilitating” in the Yerkes–Dodson sense; rather, it would reflect a statistical artifact due to the shared variance among emotional predictors. Future research using experimental designs or longitudinal data is needed to disentangle these competing explanations (Hodson and Prusaczyk, 2024).

Additionally, though this study found that listening anxiety was significantly and negatively correlated with both IELTS listening scores and self-efficacy, it has failed to uniquely predict these performances when other emotional dimensions were accounted for, suggesting that the effects of anxiety are isolated. These results resonate with Kim and Baek’s (2017) report that rather than directly impacting listening performance, listening anxiety indicated shared variance with cognitive and motivational variables. In summary, these findings imply that listening anxiety does not fully explain variance in L2 listening achievement alone, which highlights the necessity to distinguish the domain-specific emotions and general emotions and employ a skill-centered framework to assess the unique effects of each emotional dimension.

#### 5.2.2 The consistent predictive power of enjoyment and boredom

Unlike the variable and context-sensitive effects of listening anxiety, both FLLB and FLLE consistently predicted listening outcomes across different measures. Specifically, FLLE was a positive predictor of CET4 listening performance, listening self-efficacy, and IELTS listening scores, while FLLB showed negative predictive effects across all three measures. The uniform predictive relationships across the various listening assessments imply that boredom and enjoyment are robustly associated with L2 listening performance. Notably, these results build on growing evidence that positive emotions, especially enjoyment, support listening development (Yang and Wu, 2025), while also offering some of the first direct empirical support for the negative impact of listening-specific boredom.

The pattern of standardized coefficients across listening measures warrants consideration. For IELTS listening, the coefficient for FLLB was −0.408, while that for FLLE was 0.352. For CET4 listening and listening self-efficacy, the coefficients for FLLE were 0.423 and 0.394, and those for FLLB were −0.388 and −0.399. A possible reason for these numeric patterns is that the greater complexity and higher stakes of the IELTS test could elevate learners’ likelihood of disengagement, thereby strengthening the adverse influence of boredom (Li, 2021). In contrast, the relatively familiar and curriculum-integrated nature of CET4 listening tasks may promote persistent engagement and self-assurance, potentially allowing enjoyment to exert a relatively stronger influence. The findings align with Pekrun’s (2006) Control-Value Theory that enjoyment serves as an activating positive emotional dimension, while boredom functions as a deactivating negative emotional dimension. From one perspective, through the lens of this theory, enjoyment is likely to enhance listening performance by increasing motivation, strengthening attentional and cognitive ability, and expanding persistence in processing spoken input. These finding also mirrors previous L2 learning research that documented enjoyment motivates learners’ deeper involvement and sustained efforts in listening tasks (Dewaele and MacIntyre, 2014; Wang and MacIntyre, 2021). From another perspective, boredom may weaken listening performance through reducing task engagement, avoiding cognitive effort, and escalating attention removal, which are recurrently reported in L2 learning studies (Li, 2021). The strong predictive impact of both listening enjoyment and boredom on listening self-efficacy highlights the relationship between listeners’ emotions and their self-believed competence, with boredom decreasing confidence and the positive emotion increasing it in the long term.

Apart from their predictive variance, these findings bridge the conceptual and methodological gaps recognized in previous research. Listening enjoyment in previous research was measured by using or adapting general foreign language enjoyment scales, failing to clarify their validity for a listening-focused environment (Wang and MacIntyre, 2021; Yang and Wu, 2025). This study used a listening-focused measure with established reliability and validity, providing more robust support for the impact of listening-specific enjoyment on L2 listening achievement. Additionally, even though the robust negative impact of boredom across L2 learning has been highlighted in recent meta-analyses (Li et al., 2025b; Li and Yang, 2025) which called for expanding research on listening-specific boredom, empirical work on listening boredom remains scarce. Hence, this study provides evidence that listening boredom represents an important emotional impairment to L2 listening achievement.

It is worth noting that, in contrast with the more varying roles of listening anxiety, the consistent predictive effects of boredom and enjoyment imply that these two emotions are likely to be more directly connected to learners’ engagement in listening activities. While listening anxiety mostly signifies evaluation pertaining to pressure and possible failure, enjoyment and boredom are more directly related to learners’ engagement in listening activities, formed by personal relevance, perceived challenge, and task interest. This differentiation necessitates the need to elucidate the differential contributions of multiple listening-specific emotions to L2 listening achievements through a simultaneous investigation, as suggested in previous L2 studies.

Beyond their predictive effects on achievement, the interrelationships among the three listening emotions merit brief consideration. As shown in Table 7, FLLB and FLLE were moderately negatively correlated (*r* = −0.466), indicating that boredom and enjoyment are distinct but inversely related emotions. This aligns with the control-value theory (Pekrun, 2006), which conceptualizes enjoyment and boredom as distinct emotions arising from different appraisal patterns. FLLB and FLLA showed a positive correlation (*r* = 0.572), suggesting co-occurrence of negative emotions, a pattern consistent with prior research documenting that learners who experience anxiety are also prone to boredom (Li, 2021; Li et al., 2023a, 2023b; Li and Li, 2023). Conversely, FLLE and FLLA were negatively correlated (*r* = −0.544). This inverse relationship aligns with the broaden-and-build theory (Fredrickson, 2001; Dewaele and MacIntyre, 2014), which posits that positive emotions can broaden cognitive resources and buffer against the detrimental effects of negative emotional experiences. These patterns, while secondary to our main findings, underscore the complex emotional landscape of L2 listening and align with the growing body of research examining multiple emotions simultaneously (Li and Li, 2023; Pawlak et al., 2020).

### 5.3 Pedagogical implications

The results of this study highlight several key instructional considerations for teaching L2 listening. Generally, these findings highlight the importance of integrating emotional awareness into listening pedagogy, especially by nurturing enjoyment and alleviating boredom. The results indicate that these two emotions support learners’ emotional well-being while also consistently predicting listening performance. Therefore, listening instruction should not be purely cognitive-focused and should put the learner’s emotional engagement with listening activities into consideration. On the one hand, language instructors should select meaningful and engaging listening materials to cultivate students’ listening enjoyment, including integrating authentic tasks and offering encouraging feedback that reinforces students’ confidence. On the other hand, instructors may actively diminish the possibility of boredom by adjusting task difficulties, varying task formats, and providing collaborative or interactive listening activities. While the effect of listening anxiety appeared to be context-sensitive, supporting learners in sustaining optimal levels of performance pressure through strategy instruction and scaffolded support may help improve listening outcomes. Moreover, the enjoyment and boredom scales tailored to listening in this study may be used as effective diagnostic resources for evaluating learners’ emotional experiences and guiding emotion-informed instructional planning.

## 6 Conclusion

Earlier studies of L2 listening have mainly concentrated on linguistic and cognitive predictors of performance, while emotional variables have been less frequently examined, aside from anxiety. Extending prior investigations, this study examines several listening-related emotions, including anxiety, boredom, and enjoyment, and their associations with L2 listening outcomes. To accomplish this objective, listening-specific measures of foreign language listening boredom and enjoyment were developed and psychometrically validated. The empirical findings of the current study revealed that listening enjoyment significantly supported listening performance, whereas listening boredom had a significantly negative impact. On the contrary, when enjoyment and boredom were accounted for, listening anxiety showed less consistent predictive effects, which tended to be context-dependent. These findings highlight the need to incorporate listening-focused emotions into L2 listening research and psychometrically validate instruments across differing listening contexts in future research.

In spite of these significant contributions, this study is not free from limitations. The generalizability of these findings may be reduced in other learning settings and learner groups, as the population of this study was exclusively Chinese university non-English-major EFL students. Definitive causal conclusions about how listening-specific emotions relate to listening achievement can not be confirmed due to the cross-sectional nature of this study. Therefore, future research could examine listening emotions across learners with differentiated proficiency and instructional settings, or adopt longitudinal and interventional investigations. Additionally, future research could explore a broader range of listening-domain emotional experiences and examine the mediating variables in the relationship between these emotions and listening development.

## Data Availability

The raw data supporting the conclusions of this article will be made available by the authors, without undue reservation.
